# Role of Nampt and Visceral Adiposity in Esophagogastric Junction Adenocarcinoma

**DOI:** 10.1155/2017/3970605

**Published:** 2017-01-11

**Authors:** Haijun Li, E. Bai, Yong Zhang, Zhuoqi Jia, Shicai He, Junke Fu

**Affiliations:** ^1^Department of Thoracic Surgery, The First Affiliated Hospital of Xi'an Jiaotong University, Xi'an 710061, China; ^2^Department of Gynecology and Obstetrics, The First Affiliated Hospital of Xi'an Jiaotong University, Xi'an 710061, China; ^3^Department of General Surgery, The First Affiliated Hospital of Xi'an Jiaotong University, Xi'an 710061, China

## Abstract

Nampt including eNampt and iNampt may contribute to mediating obesity-associated cancers. This study investigated the role of Nampt in esophagogastric junction adenocarcinoma (EGA), a cancer strongly correlated with obesity. Visceral adiposity was defined by waist circumference or VFA. eNampt in sera were measured by enzyme-linked immunosorbent assay. iNampt expression in EGA was determined by PCR, western blot, and immunohistochemistry. Sera eNampt were significantly elevated in these overweight and obese patients, especially for viscerally obese patients, and positively correlated with BMI, waist circumference, VFA, and also primary tumor, regional lymph nodes, and TNM stage (*P* < 0.05). iNampt expression in both the mRNA and protein levels was upregulated in EGAs (*P* < 0.05). iNampt staining was found primarily in the cytoplasm and nuclei and significantly associated with tumor, lymph nodes, and TNM stage and also correlated positively with serum eNampt, BMI, total fat area, VFA, superficial fat area, and waist circumference (*P* < 0.05). iNampt, eNampt, tumor, lymph nodes, and TNM stage correlated to the survival of EGAs, and iNampt expression and TNM stage affected the prognosis independently (*P* < 0.05). This study highlighted the association of eNampt/iNampt with visceral obesity and a potential impact on the biology of EGA.

## 1. Introduction

Esophagogastric junction adenocarcinoma (EGA) is commonly regarded as a separated tumor entirety of upper digestive tract malignant tumors [1]. The incidence of EGA has been dramatically increasing in both western and eastern countries and may be associated with the elevated ratio of overweight and obese populations [2]. Tumor en bloc removal combined with perigastric-mediastinal lymphadenectomy remains a mainstream treatment for the resectable EGAs [3]. Siewert types are the well-accepted classification of EGAs, simply as the distal esophagus (type I), true carcinoma of the cardia (type II), and subcardial carcinoma (type III) [4].

Nicotinamide phosphoribosyl transferase (Nampt) includes intracellular and extracellular Nampt (iNampt and eNampt, resp.). iNampt, a pleiotropic protein, acts as the rate-limiting enzyme in the salvage pathway for NAD biosynthesis and indirectly affects both cellular energetics and NAD-dependent enzymes [5]. iNampt is involved in different immune and metabolic disorders, especially cancer. iNampt was significantly upregulated or overexpressed in the genesis of a variety of malignant tumors, such as colorectal cancer [6], breast cancer [7], and gastric cancer [8].

Obesity, a growing health problem worldwide [9], correlates to an increased risk of numerous malignancies including colon cancer and breast cancer [5]. A meta-analysis has demonstrated that the overweight and obesity are strongly related to the risk of EGA [10], and some adipokines may prompt cancer cell survival and solid tumor progression [11]. The new adipokine, eNampt, mainly secreted by visceral fat, facilitated early B cell proliferation as pre-B-cell colony-enhancing factor (PBEF) at first [12] and drew considerable interest; after that it was described as an insulin-mimetic cytokine and termed as visfatin [13]. Although all three names (Nampt, PBEF, and visfatin) have been used in publications, Nampt has been approved as the official nomenclature of the protein and the gene by both the HUGO Gene Nomenclature Committee and the Mouse Genomic Nomenclature Committee. eNampt in serum is elevated with the increase of obesity, with many roles in physiology and pathology, and may well prove to be a significant mechanistic link in the network of cytokines influencing obesity-associated tumor progression. eNampt triggers numerous intracellular signalling pathways with varying temporal dynamics and can also stimulate multiple biological effects in a variety of cell types, especially tumor cells [14].

eNampt, as an cytokine, also functions as a NAD biosynthetic enzyme similar with iNampt. In the NAD biosynthetic pathway from nicotinamide, iNampt catalyzes the transfer of a phosphoribosyl group from 5-phosphoribosyl-1-pyrophosphate to nicotinamide, forming nicotinamide mononucleotide (NMN) and pyrophosphate [15]. NMN is then converted to NAD with the help of nicotinamide mononucleotide adenylyltransferase (NMNAT) [16]. As such, these effects of eNampt can be attributed either to (i) its extracellular enzymic activity or to (ii) the binding and activation of a cell surface receptor [14]. Hence, eNampt/iNampt is so important and drew more and more interest, especially in the field of cancer. However, until now, data regarding the role of eNampt/iNampt and the association between eNampt and hematologic profile in patients with EGA is relatively limited. To investigate the role of eNampt and iNampt in EGA, we measured pretreatment plasma eNampt level as well as pretreatment hematologic profile and iNampt expression in the tumors in a Chinese population with EGA.

## 2. Materials and Methods

### 2.1. Patients and Tissues/Sera

Tumor samples and their paired adjacent nontumor tissues were collected, and the preoperative sera of 116 EGA patients were obtained from the First Affiliated Hospital of Xi'an Jiaotong University from June 2008 to May 2011. These EGA patients included 70 males and 46 females, and the median age of them was 61 years (ranging from 31 to 73 years). The study was approved by the Institutional Review Board of Xi'an Jiaotong University, and patients signed informed consent forms. All patients have not received preoperative chemotherapy or radiotherapy and have been without obvious weight loss (<5%). The patients were grouped according to gender, age, Siewert types, differentiation, tumor, lymph nodes, and TNM stage. These EGA patients were followed up over a five-year period. Disease-specific survival of EGA patients was calculated as the time from diagnosis to the date of death filtering out the effect of mortality from other causes.

### 2.2. Anthropometry

Anthropomorphic data were measured before surgery by a single observer. Waist circumference was measured to the nearest 0.5 cm at the midpoint between the lower border of the rib cage and the iliac crest following gentle expiration. Central obesity was defined as a waist circumference greater than 80 cm in women and 94 cm in men [17]. Weight was measured to the nearest 0.1 kg with the patient dressed but without shoes or heavy outerwear. Height was measured to the nearest 0.5 cm with the patient barefoot. BMI was calculated as weight/height^2^. BMI was defined utilizing the World Health Organization definitions, with a BMI of 20–24.9 kg/m^2^ (normal), 25–29.9 kg/m^2^ (overweight), and >30 kg/m^2^ (obese). All patients were asked about their body weight 12 months before diagnosis to allow an estimation of weight loss at diagnosis. Visceral fat area (VFA) and superficial fat area were calculated by computed tomography scanning of cross-sectional transverse images at the level of the third and fourth intervertebral discs [18]. The definition of visceral obesity was VFA exceeding 130 cm^2^ [19].

### 2.3. Enzyme-Linked Immunosorbent Assay

Serum eNampt level was measured utilizing commercially available eNampt ELISA kits (Linco Research, Inc., St. Charles, MO, USA) following the manufacturer's instructions in 96-well flexible microtiter plates. The wells were washed and biotinylated anti-eNampt antibody was added. After washing away unbound biotinylated antibody, HRP-conjugated streptavidin was pipetted to the wells. These wells were washed again, and a TMB substrate solution was used as the detecting agent. The OD of each well was read at 450 nm.

### 2.4. RNA Expression Study

Total RNA was isolated from tissue utilizing the TRIzol reagent (Invitrogen, San Diego, CA) and then reverse-transcribed to cDNA with a cDNA synthesis kit (Takara Biochemicals, Japan) according to the manufacturer's protocols. Expression of* Nampt* mRNA was quantified by RT-PCR, and transcript levels were normalized to *β-actin*. Briefly, the reaction was run using an Icycler (Bio-Rad, Hercules, CA) with a preheating step at 95°C for 10 min, followed by 30 cycles at 94.0°C for 30 sec, 55.0°C for 30 sec, and 72°C for 1 min. The primers used in the reaction were as follows: *β-actin*, forward: 5′-ATCGTGCGTGACATTAAGGAGAAG-3′, reverse: 5′-AGGAAGGAAGGCTGGAAGAGTG-3′;* Nampt*, forward: 5′-AAGAGACTGCTGGCATAGGA-3′, reverse: 5′-ACCACAGATACAGGCACTGA-3′. All cDNA samples were synthesized in parallel, and PCR reactions (the product, *β-actin* 179 bp;* Nampt* 181 bp) were run in triplicate.

### 2.5. Protein Extraction and Western Blotting

Protein from tissue was extracted with RIPA buffer, and the concentration was quantified via the BCA method (Pierce). An equal amount of protein was separated by SDS-PAGE and then transferred onto PDVF membranes (Millipore). Western blot analyses were then performed using anti-iNampt antibody (Santa Cruz Biotechnology, Santa Cruz, CA) or an anti-*β*-actin antibody (Santa Cruz Biotechnology). The blots were developed with chemiluminescence substrate solution from Pierce and exposed to X-ray film.

### 2.6. Immunohistochemistry (IHC)

IHC was carried out on paraformaldehyde-fixed paraffin sections. Primary anti-iNampt antibody (Santa Cruz Biotechnology) was used in the IHC with streptavidin peroxidase (SP) conjugated method. IHC was performed and the staining results for iNampt were semiquantitatively analyzed as previously reported [20], and sections with a total score of >4 were defined as exhibiting positive staining for iNampt. All histological analyses were carried out by three independent observers.

### 2.7. Statistical Analysis

Values were presented as the mean ± SD. Continuous variables were compared using unpaired *t*-tests for normally distributed data and Mann–Whitney *U* test otherwise. Association of categorical variables was assessed using *χ*^2^ test. Correlations between variables were assessed using the Spearman and Pearson correlation coefficients, as appropriate. Survival curves were estimated by the Kaplan-Meier method. Cox's proportional hazards regression analysis was done to estimate which factors might have a significant influence on survival. *P* < 0.05 was considered statistically significant.

## 3. Results

### 3.1. eNampt Level in the Serum

Serum level of eNampt measured using ELISA correlated with obesity status and pathologic phase. Mean eNampt level was significantly higher in the serum of patients who were defined as obese by BMI (7.81 ± 1.54 versus 6.43 ± 1.96 ng/ml for BMI 25 kg/m^2^ or greater versus less than 25 kg/m^2^, *P* < 0.001), waist circumference (7.82 ± 1.36 versus 6.50 ± 2.02 ng/ml, *P* < 0.001), and VFA (8.00 ± 1.45 versus 5.84 ± 1.75 ng/ml, *P* < 0.001). eNampt level correlated with BMI (*r*_*s*_ = 0.34, *P* < 0.001), waist circumference (*r*_*s*_ = 0.50, *P* < 0.001), and VFA (*r*_*s*_ = 0.54, *P* < 0.001); therefore VFA was the most close factor associated with eNampt.

Circulating eNampt level also correlated with primary tumor (*r*_*s*_ = 0.37, *P* < 0.001), regional lymph nodes (*r*_*s*_ = 0.33, *P* < 0.001), and TNM stage (*r*_*s*_ = 0.42, *P* < 0.001) but not with other clinicopathological factors including gender, age, Siewert types, and tumor differentiation (*P* > 0.05). Median disease-specific survival was significantly different between patients with the level of eNampt below and above the median (36.00 ± 0.69 versus 32.00 ± 1.04 months, *P* = 0.002).

### 3.2. Expression of iNampt in EGA Tissues and Clinicopathological Characteristics

**Figure 1:**
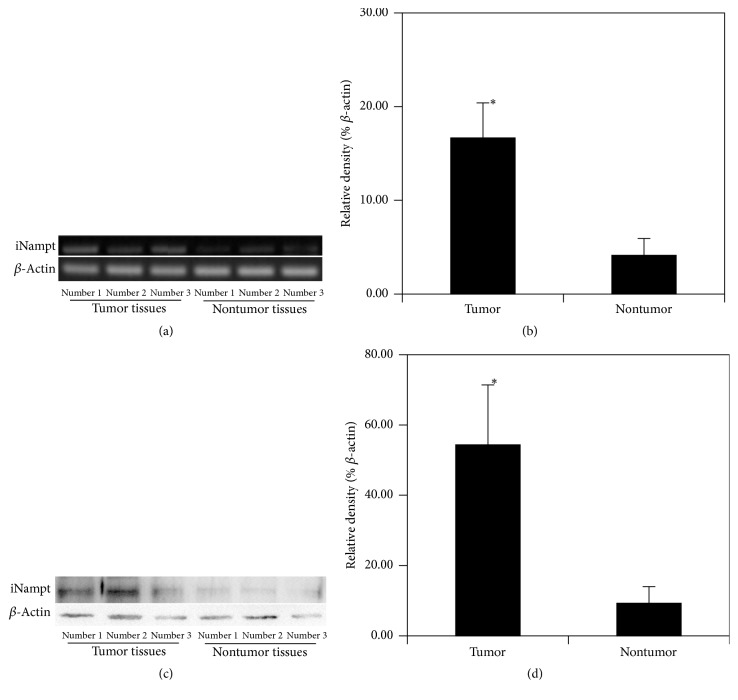
iNampt expression in EGAs tissues and their adjacent nontumor tissues (*n* = 5; 3 of 5 representative images were shown). Compared to these adjacent nontumor specimens, EGA had upregulated iNampt expression in the mRNA level ((a) and (b), 16.66 ± 3.74% versus 4.14 ± 1.79%, ^*∗*^*P* = 0.001 < 0.05) and protein level ((c) and (d), 54.34 ± 17.05% versus 9.32 ± 4.69%, ^*∗*^*P* = 0.003 < 0.05).

Expression of iNampt in EGAs and their adjacent nontumor tissues was analyzed in the mRNA and protein levels. Compared to their adjacent nontumor specimens, EGAs tissues had higher iNampt expression in both the mRNA and protein levels regardless of the background of obesity (Figure 1).

**Figure 2:**
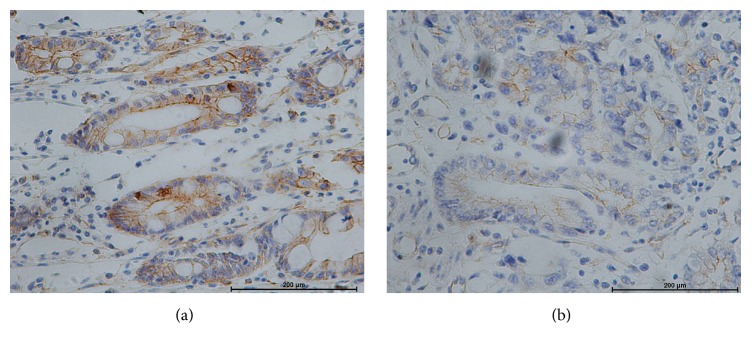
The protein expression of iNampt in these EGA specimens was analyzed by immunohistochemical staining (×400). iNampt expression was significantly upregulated in these tumors from obese patients, especially visceral obesity (a), compared to those nonobese patients (b) (7.31 ± 3.04 versus 4.34 ± 2.64, *P* < 0.001).

iNampt protein expression was also assessed semiqualitatively by immunohistochemical analysis. Immunoreactivity for iNampt was found primarily in the cytoplasm and nuclei (Figure 2). Expression of iNampt was significantly associated with tumor, lymph nodes, and TNM stage (Table 1, *P* < 0.05). However, there were no correlations to gender, age, Siewert types, and tumor differentiation (*P* > 0.05). iNampt protein expression in these tumors was positively correlated with sera eNampt of these patients (*r* = 0.80, *P* < 0.001). There was a higher proportion of patients with lymphatic invasion in those with greater than median iNampt expression (79.69 versus 25.00%, *P* < 0.001).

### 3.3. iNampt Protein Expression in the Tumors and Their Obesity Status

iNampt expression in those tumor specimens from EGAs was also evaluated in consideration of metabolic features. iNampt expression correlated moderately with all markers of obesity (Table 2). Patients in the highest iNampt expression quartile had significantly higher BMI (*P* = 0.018), wider total fat area (*P* < 0.001), larger VFA (*P* < 0.001), bigger superficial fat area (*P* = 0.004), and upper waist circumference (*P* < 0.001) than those in the lowest quartile (Table 3). Viscerally obese patients were 4.1 times more likely to be within the highest quartile of iNampt expression than the lowest (*χ*^2^ = 19.66, *P* < 0.001). Patients classified as viscerally obese had significantly higher tumor iNampt expression than patients who were not viscerally obese (7.31 ± 3.04 versus 4.34 ± 2.64, *P* < 0.001).

### 3.4. Nampt, Obesity Status, Clinicopathological Features, and Survival

**Figure 3:**
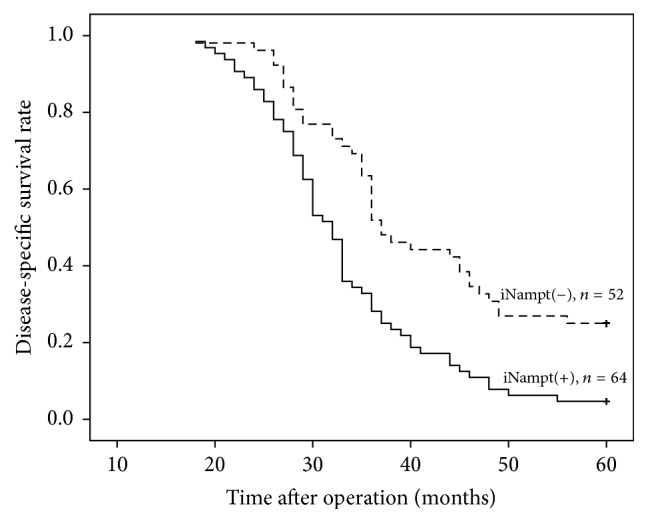
Kaplan-Meier analysis of disease-specific survival of patients with EGA relative to iNampt expression.

Disease-specific survival was significantly reduced in those with positive compared with negative iNampt staining (33.59 ± 1.21 versus 41.85 ± 1.76 months, *P* < 0.001, Figure 3). On univariable regression analysis, iNampt (positive versus negative expression), eNampt (below versus above the median), tumor (T1 and T2 versus T3 and T4), lymph nodes (N0 versus N1 and N2), and TNM stage (I and II versus III and IV) correlated to the survival of EGAs, but BMI, waist circumference, and VFA were not related to the prognosis of these patients with EGA. Positive tumor iNampt staining was associated with death from EGA compared with iNampt-negative tumors (HR = 6.78, *P* < 0.001), and TNM stages I and II were associated with death from EGA compared with stages III and IV (HR = 0.06, *P* < 0.001). On multivariable analysis, both iNampt expression and TNM stage were independently associated with the survival (Table 4).

## 4. Discussion

A variety of clinical studies have linked adiposity with increased cancer incidence, progression, and metastasis, and adipose tissue is now being credited with both systemic and local effects on tumor development and survival [21]. Elucidating the mechanisms linking obesity and EGA is so complex by the numerous influences of obesity. Obesity primarily manifests the accumulation of adipose tissue. Adipose tissue was regarded as an insulating and mechanically supportive site of energy storage for a long time and now draws more and more attention for secreting a variety of adipokines [22]. Adipokines are polypeptide growth factors and cytokines and are secreted mainly by white adipose tissue preadipocytes and mature adipocytes. These potent adipokines such as resistin, leptin, and adiponectin are involved in cell growth, proliferation, cell cycle control, and angiogenesis [23] and could play a significant role in facilitating tumor growth and metastasis.

The new adipokine, eNampt, is not only produced by adipocytes and pre-B-cells but also readily detectable in conditioned media from cultures of most cell types, including cancer cells, such as hepatoma cells, colorectal cancer cells, breast cancer cells, melanoma cells, prostate cancer cells, and cervical cancer cells [14]. eNampt acts as a cytokine and demonstrates its biological potential as a putative paracrine and autocrine factor. eNampt may promote tumor progression in a large number of cancers including breast cancer [24, 25] and prostate cancer [26]. Furthermore, eNampt levels in sera were associated with tumor progression in malignant astrocytomas [27], gastric cancer [28], and colorectal cancer [29]. This study demonstrated the close relationship between EGA and eNampt and showed that eNampt was significantly associated with tumor, regional lymph nodes, and TNM stage, indicating that EGA cells released eNampt into the sera. This study also revealed that eNampt was significantly correlated to these markers of obesity including BMI, waist circumference, and VFA, indicating that adipose tissue secreted eNampt into the sera. These results were consistent with the above-mentioned notion. eNampt provided by these two sources and others might recognize some cell surface receptor and also acts as a NAD biosynthetic enzyme [14]. As such, eNampt may promote EGA development by increasing intracellular NAD^+^ content, which directly affects the ability of Sirt1 or by other ways.

In addition, iNampt is closely related to malignant tumors including gastric cancer [8] and was also overexpressed in the EGA specimens in this study. This study also showed that expression of iNampt was significantly associated with tumor, regional lymph nodes, and TNM stage but not correlated with gender, age, Siewert types, and tumor differentiation. It was very interesting in this study that iNampt expression seems to be age-dependent. Further analysis showed that more patients who were older than 60 years were viscerally obese compared to others (39/66 versus 19/50, *P* < 0.05), although these two groups had no difference in the BMI and waist, and the expression of iNampt in EGA tissues was not significantly correlated with age of patients (*r*_*s*_ = 0.17, *P* = 0.166).

The novel findings of this study that serum eNampt were positively correlated with the expression of iNampt in the tumors were consistent with the notion that changed adipokines and other growth factors following obesity formation may promote cancer cell survival and solid tumor progression [30]. In this study, the altered iNampt expression was associated with changes in disease-specific survival. iNampt was correlated positively with serum eNampt, and eNampt were significantly elevated in the sera of overweight and obese patients (BMI > 25 kg/m^2^, which was defined as obese in china), especially for viscerally obese patients, and positively correlated with BMI, waist circumference, and VFA. Hence, iNampt was associated with all markers of obesity including BMI, total fat area, VFA, superficial fat area, and waist circumference. The fact that median disease-specific survival was significantly longer in these patients below the median eNampt level than above that was consistent with the results that survival was shorter in those with positive iNampt staining than negative expression. This study supports the hypothesis that obesity status influences eNampt/iNampt expression, but it is noticeable that all markers of obesity were not correlated to the survival of these patients in the study.

For valid evidence, the patient cohort was very restricted to those undergoing surgical treatment with curative intent and without obvious weight loss in this study. iNampt, eNampt, tumor, regional lymph nodes, and TNM stage correlated to the survival of EGAs, and iNampt expression and TNM stage were independently associated with the survival. Hence, further studies are required to determine the downstream impact of this altered eNampt and iNampt expression on the pathways that impact survival, proliferation, and apoptosis.

## 5. Conclusion

iNampt expression was independent prognostic indicator for EGA patients. This study highlighted the association of eNampt/iNampt with visceral obesity and a potential impact on the biology of EGA. Targeting eNampt/iNampt may have a rationale in future studies.

## Figures and Tables

**Table 1:** Clinical correlation of iNampt protein expression in EGA.

Clinicopathological features	Categories	iNampt expression		*χ* ^2^	*P*
		−	+		
Gender	Male Female	31 21	39 25	0.21	0.89
Age (years)	<60 ≥60	27 25	23 41	2.99	0.08
Siewert types	I II III	8 27 17	19 26 19	3.41	0.18
Differentiation	G1 & G2G3 & G4	25 27	33 31	0.14	0.71
Tumor	T1 & T2T3 & T4	38 14	25 39	13.38	<0.001^*∗*^
Lymph nodes	N0 N1 & N2	39 13	13 51	34.69	<0.001^*∗*^
TNM stage	I II III	18 26 8	4 16 44	35.35	<0.001^*∗*^

TNM: tumor node metastasis.

^*∗*^Statistically significant.

**Table 2:** Correlations between relative quantification values for iNampt and measures of obesity.

Measures of obesity	iNampt expression	
	*r*_*s*_	*P*
Body mass index	0.25	0.007^*∗*^
Waist circumference	0.40	<0.001^*∗*^
Visceral fat area	0.49	<0.001^*∗*^
Superficial fat area	0.33	<0.001^*∗*^
Total fat area	0.46	<0.001^*∗*^

^*∗*^Statistically significant.

**Table 3:** Differences in obesity status between tumors with lowest- versus highest-quartile iNampt expression.

Obesity status	iNampt expression		*P*
	Lowest-quartile	Highest-quartile	
Body mass index (kg/m^2^)	24.00 ± 1.76	25.38 ± 2.26	0.018^*∗*^
Waist circumference (cm)	81.26 ± 4.01	87.87 ± 4.45	<0.001^*∗*^
Total fat area (cm^2^)	184.70 ± 22.16	215.56 ± 17.11	<0.001^*∗*^
Visceral fat area (cm^2^)	118.50 ± 14.62	141.18 ± 9.65	<0.001^*∗*^
Superficial fat area (cm^2^)	66.20 ± 9.74	74.39 ± 9.78	0.004^*∗*^

^*∗*^Statistically significant.

**Table 4:** Cox regression analysis of factors associated with death from EGA.

	Univariable analysis		Multivariable analysis	
	HR (95% CI)	*P*	HR (95% CI)	*P*
iNampt (positive versus negative expression)	6.78 (1.81–25.33)	0.002^*∗*^	3.00 (1.03–8.69)	0.043^*∗*^
eNampt (below versus above the median)	0.15 (0.04–0.55)	0.002^*∗*^	0.38 (0.14–1.01)	0.052
Tumor (T1 & T2 versus T3 & T4)	0.14 (0.03–0.64)	0.004^*∗*^	0.76 (0.46–1.25)	0.759
Lymph nodes (N0 versus N1 & N2)	0.09 (0.02–0.41)	<0.001^*∗*^	0.63 (0.34–1.19)	0.156
TNM stage (I & II versus III & IV)	0.06 (0.01–0.46)	<0.001^*∗*^	0.20 (0.09–0.43)	<0.001^*∗*^

TNM: tumor node metastasis.

^*∗*^Statistically significant.
